# Skin Diseases and Their Remedies

**Published:** 1860-11

**Authors:** 


ART. XI.—*Skin Diseases and their Remedies.* By ROBERT J. JORDAN, M.D. 12mo.; pp. 283. London, 1860.

THE work of Dr. Jordan does not admit of any analysis, for it is itself an analysis of a most important class of diseases and of their remedies. We look upon the work as a most opportune one, and have no doubt it will meet with an extensive circulation both in Great Britain and in this country. Comprised within an extremely small compass, in comparison with the great treatises of Alibert, Rayer, Wilson, and others, it is sufficiently full for all the purposes of the student and the young practitioner, who may safely refer to it as a faithful guide to what they will be likely to meet with in their daily attendance at the hospital and at the bedside of the sick in private practice. The classification of the author impresses us most favorably; his descriptions are exceedingly clear and definite; and his treatment, in most instances, is remarkably judicious. We hope that the work will soon pass to another edition, and that it may be speedily republished in this country, where, we are quite certain, it would be well received.

